# Low-frequency magnetic response of gold nanoparticles

**DOI:** 10.1038/s41598-023-48813-y

**Published:** 2023-12-07

**Authors:** Saba Harke, Atefeh Habibpourmoghadam, Andrey B. Evlyukhin, Antonio Calà Lesina, Boris N. Chichkov

**Affiliations:** 1 Institute of Quantum Optics, Leibniz University Hannover, Hannover, 30167 Germany; 2 Cluster of Excellence PhoenixD, Leibniz University Hannover, Hannover, 30167 Germany; 3 Hannover Centre for Optical Technologies, Leibniz University Hannover, Hannover, 30167 Germany; 4 Lower Saxony Centre for Biomedical Engineering, Implant Research and Development, Hannover, Germany; 5 Institute for Transport and Automation Technology, Leibniz University Hannover, Hannover, 30167 Germany

**Keywords:** Biomedical engineering, Bionanoelectronics, Nanoparticles

## Abstract

Gold nanoparticles (AuNPs) exposed to low frequency magnetic fields have shown promise in enhancing biological processes, such as cellular reprogramming. Despite the experimental evidence, a comprehensive understanding of the underlying physical principles and the corresponding theory remains elusive. The most common hypothesis is that functionalized nanoparticles transiently amplify magnetic fields, leading to improved cellular reprogramming efficiency. However, a detailed investigation on this topic is lacking. This paper bridges this knowledge gap by conducting a comprehensive investigation on the magnetic response of surface-modified AuNPs exposed to magnetic fields with frequencies up to hundreds of MHz. Starting with the inherent properties of bulk gold material, we explore a wide range of magnetic susceptibilities that might result from the redistribution of charge carriers due to bond molecules on the particle surfaces. Through analytical models and numerical electromagnetic simulations, we examine various geometric factors that can enhance the magnetic response, including the number of particles, spatial distribution, size, and shape. Our broad investigation provides researchers with analytical and numerical estimates of the magnetic response of nanoparticles, and the associated limits that can be expected. We found that a magnetic field enhancement comparable to the incident field requires very high magnetic susceptibilities, well beyond the values measured in functionalized gold nanoparticles thus far.

## Introduction

Metallic nanoparticles have shown promising potential in various biomedical applications, such as drug delivery^1,2^, imaging^3–5^, and hyperthermia therapy^6–9^. Compared to bigger particles, nanoparticles have a high surface area to volume ratio, which makes them highly reactive to bind to other molecules^10^. Gold nanoparticles (AuNPs), specifically, have gained attention due to their unique optical^11,12^ and chemical^13^ properties with high biocompatibility^14,15^. In the optical frequency regime AuNPs exhibit localized surface plasmon resonances due to the collective oscillation of free electrons in response to an electromagnetic field. Furthermore, AuNPs possess antibacterial properties^16^ and can catalyze chemical reactions due to their unique electronic properties^17^. For the use of electromagnetic fields (EMFs) at high frequencies (up to the visible regime) in combination with AuNPs, different physical mechanisms of interaction were already determined like radio-frequency EMFs heat-induction (as used for ablation of cancer cells)^18,19^ and light-induced Raman-active vibrations (as used for the detection of cells)^20^. These and many more mechanisms of action, as heat-induction due to thermoplasmonics^21^ and phothermal therapy^18,19,22^ are also focuses of current research.

In the low frequency regime studies including AuNPs do usually not address the physical interaction mechanism between nanoparticles and magnetic fields^23–27^. However, there are hypotheses that the presence of nanoparticles causes relevant magnetic field alterations, such as changes in local field distribution or field enhancement, which lead to enhancement of biological responses^28–31^. Remarkably, application of low frequency magnetic fields with AuNPs has been shown to enhance conversion efficiency in cellular reprogramming^28^. In this regard^28^, mouse fibroblasts were cultivated on a substrate of AuNPs and treated with magnetic fields in the presence of transient reprogramming factors to mediate their direct lineage reprogramming into induced dopamine neurons. The authors found a significantly increased expression of neuronal marker genes and a 20-fold increase in conversion efficiency compared to the controls. In another study^29^ AuNPs were injected in-vivo into the hippocampus of mice and stimulated high-efficient neurogenesis by exposure to magnetic fields, confirming the beneficial effect of AuNPs during magnetic field treatment. The authors in both studies^28,29^ hypothesize that significant transient magnetization of the particles is causing the biological reactions. This theory is also taken up by other researchers^30^. Besides for cellular reprogramming and neurogenesis, AuNPs and low frequency magnetic fields were also applied for cell proliferation^31^ and controllable drug release^27^. However, the magnetic response of AuNPs to low-frequency magnetic fields, particularly the influence of nanoparticle size, shape, number, and electromagnetic properties, is yet to be fully explored.

Plain bulk gold material is diamagnetic with a volume magnetic susceptibility of $\chi _{\text{p}} = -3.4\times 10^{-5}$, while $\mu _{\text{r,p}} = 1+\chi _{\text{p}}$ is the relative magnetic permeability^32^. Depending on their size, shape and surface modifications, the magnetic susceptibilities of AuNPs are known to differ significantly from that of bulk gold. At the nanoscale, magnetic phenomena are influenced by both volume and surface effects, which modify the electronic structure of materials. While in bulk metals charge carriers can move steadily through energy states, there is a transfer towards discrete energy states (due to the quantum confinement effects) in nanoparticles^33^. New magnetic properties arise due to the geometric confinement of electrons and the large fraction of surface atoms present in nanoparticles^33^. Thereby, surface charges can be transferred to the inner part of the material due to energy minimization and imbalanced spins of charges near the surface of nanoparticles can appear. The imbalanced spins can cause additional magnetic moments^34^.

While the magnetic properties of plain AuNPs can already be different compared to bulk gold material, in surface modified particles the binding of molecules can cause significant additional alterations of magnetic properties. This changes are caused by rearrangement of charge carriers due to the surface modifications^33,35,36^. Since the surface of a particle is the primarily affected region, the significance of the bond particles in terms of change of magnetic properties decreases for bigger particle sizes with lower surface to volume ratios. Surface modified AuNPs can obtain stronger diamagnetic material properties than bulk gold material and even para- and ferromagnetic properties^36–39^. In thiol-capped AuNPs an appearance of magnetic moment is associated with charge transfer from the capping molecules^33,35^. Thiolated AuNPs can have hysteresis magnetization curves with remnant magnetization and coercitive field^40,41^. The details of physical interplay of the surface atoms of AuNPs with the (very different) binding partners are yet to be explained. Furthermore, relations to the sizes of the AuNPs need to be generally specified. In this regard and to the best of our knowledge, there is no theory with scientific consensus which allows a prediction of the magnetic properties of AuNPs due to surface modification. Broad and detailed overviews over the different experimental findings and formulations of possible theories explaining the experimental results are provided elsewhere^38,39^. The lack of detailed knowledge makes it difficult to quantify the limits of possible magnetic susceptibility values for surface modified AuNPs. Some empirically determined values in different studies are as follows: the magnetization curves of dodecanethiol-capped AuNPs^42^ reveal a mass magnetic susceptibility $\chi {{_\text{p,mass}}} = \chi {{_\text{p}}}/\rho$ ($\rho$ being the density and $\chi {{_\text{p}}}$ being the volume magnetic susceptibility) in the range of ${\chi {{_\text{p,mass}}} = 5\cdot 10^{-6}}\,{\mathrm{m^3/kg}}$ at a temperature of ${T=300\,\text{K}}$, while the magnetic susceptibility increases even further at lower temperatures. For comparison: the mass magnetic susceptibility of bulk gold is not only negative in sign but also at least three orders of magnitude smaller (approximately ${\chi {{_\text{p,mass}}} = -1.76\times 10^{-9}}\,{\mathrm{m^3/kg}}$)^33,43^. An example of a diamagnetic response is provided for ellipsoidal thiolated polyethylene glycol AuNPs where the volume magnetic susceptibility is determined as ${\chi _{\text{p}} = -4.9\times 10^{-4}}$, which is one order of magnitude higher than the susceptibility of bulk gold material^20^. Besides the surface modification, it is speculated that the shape of the particle plays a determining role for the magnetic susceptibility of a particle^37^.

In this theoretical study, we investigate the response of AuNPs with various characteristics (such as size, number, magnetic susceptibility and shape) to sinusoidal external magnetic fields with frequencies up to hundreds of Megahertz. We identify analytical formulas to obtain the magnetic response of AuNPs. The applicability of the formulas are validated with numerical electromagnetic simulations. We provide a comprehensive methodology for researchers to approximate the magnetic response they can expect when using AuNPs. Furthermore, we show the limits of magnetic response that can be expected from functionalized AuNPs with an example.

In the first section, starting from spherical particles with bulk gold material properties, we determine the transition of the stationary magnetic response to the dynamic magnetic response in dependence of the applied frequency and the particle size. We validate the applicability of the analytical formulas for the calculation of the magnetic response with numerical electromagnetic simulations. Furthermore, we show the magnetic field distribution as well as the gradient of the magnetic field around an individual particle and we show the impact of the particle size on both quantities. These considerations serve as a fundament for subsequent investigations considering different magnetic susceptibilities within ${-0.9\le \chi _{\text{p}}\le 10}$. Additionally, we investigate the interaction of two neighboring spherical particles as a function of their magnetic susceptibility which serves as a starting point for an investigation of the interaction of several particles. Moving on to the second section, we first examine the magnetic field response of individual prolate and oblate elliptical particles in relation to that of spherical nanoparticles. This allows us to compare the interaction behavior of the differently shaped particles with each other. To explore possible limits of magnetic field response, we determine the configurations of AuNPs that result in maximum magnetic field enhancement. In the third section, we put emphasis on an example of a diamagnetic array of particles used during cell culture experiments^28^. We exemplary consider arrays of spherical AuNPs and investigate their magnetic responses. We investigate possible limits of magnetic field responses in dependence on distribution of the particles relative to the incident magnetic field. Finally, we present a discussion of the findings from all our investigations.

## Results

### Magnetic response of spheres

To gain a better understanding of the response of a gold nanoparticle to an external sinusoidal magnetic field relevant parameters must be determined. This can be achieved through the use of analytical expressions, which allow for a structured and quantitative assessment of the relationship between the parameters and response. To this end, we present the applicability of analytical solutions for the magnetic field distribution of a spherical particle, which will serve as a foundation for our subsequent analyses. Also as a basis for subsequent investigations, we show the magnetic response of an individual AuNP with bulk gold material properties and investigate the effect of the particle size on the magnetic response. Finally, we consider the interaction of two spherical particles as a function of their magnetic susceptibilities (${-0.9\le \chi _{\text{p}}\le 10}$) as this provides the basis of both: the investigation of interaction of different shapes of particles and the investigation of interaction of several particles.

#### Particle polarizabilities

The magnetic dipole moment vector $\textbf{m}$ of a spherical particle which is exposed to an incident magnetic field $\textbf{H}{_{\text{inc}}}$ can be expressed with two magnetic polarizability contributions: $\alpha _{\text{m}}$ and $\alpha _\text{e}$. $\alpha _\text{m}$ is the magnetic polarizability due to magnetization of the particle, and $\alpha _\text{e}$ is the magnetic polarizability due to conductive and displacement currents within the particle (details on the calculations can be found in the methods section):

1 $$\begin{aligned} \textbf{m} = (\alpha _\text{e}+\alpha _\text{m})\textbf{H}{_\text{inc}} . \end{aligned}$$

The experimental studies described in the introduction were conducted at frequencies ${f < 1 {\text{MHz}}}$ ^23–31^. Since we will consider nanoparticles with sizes not exceeding several tens of nanometers, the wavelength of the external field is much bigger than the considered particles and $R{{_\text{p}}}k_0 \ll 1$, where $R{{_\text{p}}}$ is the particle radius and $k_0$ is the wavenumber in free space. Under these conditions, for calculation of $\alpha _{\text{m}}$, the quasi-static approximation^44^ can be applied leading to the following expression^45^

2 $$\begin{aligned} \alpha _{\text{m}} = \frac{3V\chi _{\text{p}}}{3+\chi _{\text{p}}} , \end{aligned}$$

where $\chi _{\text{p}}$ is the volume magnetic susceptibility with the relative magnetic permeability being $\mu _{\text{r}}=1+\chi _{\text{p}}$ and $V=4\pi R_p^3/3$ is the volume of the particle. In the quasi-static approximation (negligible retardation), with $R{{_\text{p}}}k_0 \ll 1$, $\alpha _\text{e}$ can be expressed with^46,47^

3 $$\begin{aligned} {\alpha _\text{e}} = \frac{V{R_\text{p}^2}k^2}{10} . \end{aligned}$$

$k= k_0 \sqrt{\varepsilon _{\text{p}}}$ is the wavenumber in the magnetized volume *V*, and $\varepsilon _{\text{p}}=\varepsilon _{\text{p,r}}+{\text{i}}{\sigma _{\text{p}}}/{(2\pi f\varepsilon _0)}$ is the relative permittivity of the particle accounting the dielectric part contribution $\varepsilon _{\text{p,r}}$ and the specific conductivity $\sigma _{\text{p}}$. $\varepsilon _0$ is the vacuum dielectric constant, $\mathrm {i}$ is the imaginary unit. Equations (2) and (3), show that while $\alpha _\text{m}$ is independent of frequency, $\alpha _\text{e}$ is frequency-dependent due to the wave number dependence. The ratio between the two polarizabilities can be presented as

4 $$\begin{aligned} \frac{{\alpha _\text{e}}}{\alpha _{\text{m}}} = \frac{{R_\text{p}^2}k_0^2{\varepsilon _{\text{p}}}(3+\chi _{\text{p}})}{30\chi _{\text{p}}} . \end{aligned}$$

**Figure 1:**
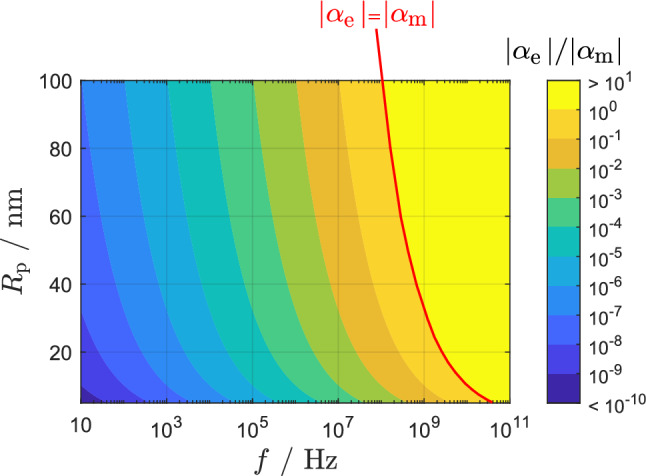
Size- and frequency dependent ratio of the two magnetic polarizability contributions in an AuNP with bulk gold material properties.

Since ${\alpha _\text{e}}/{\alpha _\text{m}}\sim {{R_ {\text{p}}^2}k_0^2}$, it can be expected that in the low-frequency range the main mechanism of interaction of gold particles with an external electromagnetic field is their quasi-static magnetization. Figure 1 illustrates the ratio of the magnitudes of the two magnetic polarizability contributions, $|{\alpha _\text{e}}| / |{\alpha _\text{m}}|$, as a function of frequency *f* and particle radius $R{_\text{p}}$. To model the behavior of a spherical gold particle, we used the material parameters for bulk gold, specifically the electrical conductivity $\sigma _{\text{p}}=41\,\mathrm{MS/m}$, the relative permittivity $\varepsilon _{\text{r,p}}=1$, and the magnetic susceptibility $\chi _{\text{p}}=-3.4\times 10^{-5}$. Figure 1 shows that for ${R{{_\text{p}}} \le 100\,{\text{nm} }}$ and ${f \le 100\,{\text{MHz}}}$, $|{\alpha _ {\text{m}}}|\ge |{\alpha _ {\text{e}}}|$. Since the experimental studies described in the introduction were conducted at frequencies ${f < 1 {\text{MHz}}}$^23–31^, we consider in this article that $|{\alpha _ {\text{m}}}|\gg |{\alpha _ {\text{e}}}|$, and the magnetic dipole moment induced in the nanoparticle can be obtained with the static approximation $\textbf{m} = \alpha _\text{m}{} \textbf{H}{_\text{inc}}$, neglecting a contribution of the dynamic response. Note that the analytically obtained result presented in Fig. 1 has been validated with numerical electromagnetic simulations in Comsol Multiphysics (details on the simulations are presented in the “Methods” section).

#### Response of a single particle with bulk gold material properties

In the static approximation, the expression for the magnetic field outside a spherical particle with magnetic dipole moment $\textbf{m}$ is

5 $$\begin{aligned} {\textbf{H}} = {\frac{1}{4\pi }\left( -\frac{{\textbf{m}}}{r^3}+\frac{3({\textbf{m}}\,{\textbf{r}}){\textbf{r}}}{r^5}\right) + \textbf{H}_{\text{inc}}} = \frac{1}{4\pi } \frac{3V\chi _{\text{p}}}{3+\chi _{\text{p}}} \left( -\frac{{\textbf{H}_{\text{inc}}}}{r^3} + \frac{3({\textbf{H}_{\text{inc}}}\,{\textbf{r}}){\textbf{r}}}{r^5}\right) + \textbf{H}_{\text{inc}}. \end{aligned}$$

where $\textbf{r}$ represents the vector connecting the center of the sphere and the point at which the magnetic field is being evaluated. With (5), the following equations can be obtained

6a $$\begin{aligned} {\textbf{H}}_\parallel&= \frac{1}{2\pi } \frac{3V\chi _{\text{p}}}{3+\chi _{\text{p}}} \frac{{\textbf{H}_{\text{inc}}}}{r^3} + \textbf{H}_{\text{inc}} \quad \text { for }\quad \textbf{r} \parallel \textbf{H}_{\text{inc}} , \end{aligned}$$

6b $$\begin{aligned} {\textbf{H}}_\perp&= -\frac{1}{4\pi } \frac{3V\chi _{\text{p}}}{3+\chi _{\text{p}}} \frac{{\textbf{H}_{\text{inc}}}}{r^3}+ \textbf{H}_{\text{inc}} \quad \text { for }\quad \textbf{r} \perp \textbf{H}_{\text{inc}} . \end{aligned}$$

When considering this formulation from (5) it becomes clear that depending on the magnetic susceptibility $\chi _{\text{p}}$ and the angle of $\textbf{r}$ relative to $\textbf{H}_{\text{inc}}$, the magnetic field $\textbf{H}$ can be enhanced or lowered compared to the incident field [note the different signs in the Eq. (5)].

The magnetic field inside the sphere can be calculated with^45^

7 $$\begin{aligned} {\textbf{H}} = {-\frac{\chi _{\text{p}}}{3+\chi _{\text{p}}}{} \textbf{H}_{\text{inc}}+\textbf{H}_{\text{inc}}} = \frac{3}{3+\chi _{\text{p}}}{} \textbf{H}_{\text{inc}}. \end{aligned}$$

**Figure 2:**
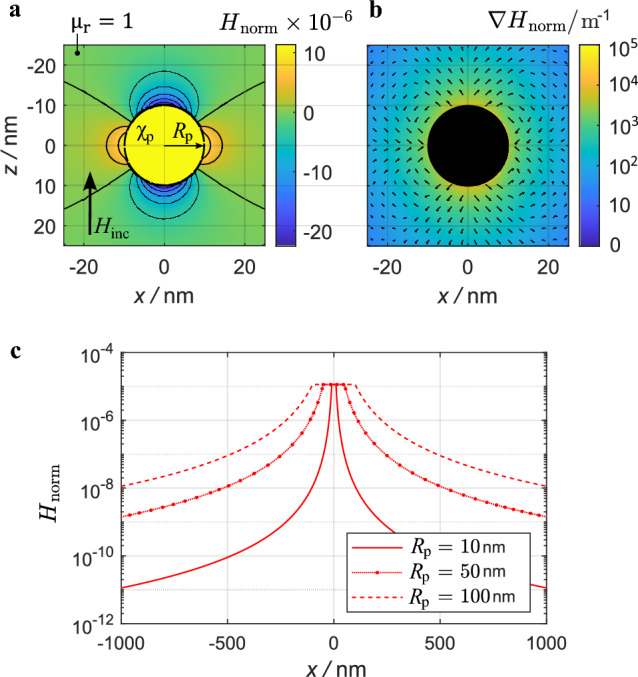
(**a**) Secondary magnetic field ${H{_\text{norm}} = (|\textbf{H}|-|\textbf{H}{_\text{inc}}|)/|\textbf{H}{_\text{inc}}}|$ and (**b**) its gradient. Both plots in the *xz*-plane. (**c**) ${H{_\text{norm}}}$ along the *x*-axis for different AuNP sizes. All plots for bulk gold material properties.

Results in Fig. 2 were calculated for an AuNP with radius ${R{{_\text{p}}} = 10\,\text{nm}}$ that is exposed to $\textbf{H}_{\text{inc}}$ directed along the *z*-axis of the Cartesian coordinate system with the origin at the particle center. Figure 2a illustrates the normalized change of the magnitude of the total magnetic field with respect to the incident field: ${H{_\text{norm}} = (|\textbf{H}|-|\textbf{H}{_\text{inc}}|)/|\textbf{H}{_\text{inc}}}|$ ($|\textbf{H}|$ being the total magnetic field magnitude). By applying this definition, the magnitude of $H{_\text{norm}}$ shows the relative change in $\textbf{H}$ compared to $\textbf{H}{_\text{inc}}$ due to the particle and the sign of $H{_\text{norm}}$ indicates whether $\textbf{H}$ is lower or higher than $\textbf{H}{_\text{inc}}$. Within this article $H{_\text{norm}}$ is referred to as the secondary magnetic field. In Fig. 2a the diamagnetic AuNP shows maximum enhancement of the secondary magnetic field where the incident magnetic field is oriented tangential to the surface of the sphere, and the secondary magnetic field is weakest where the incident magnetic field is oriented perpendicularly relative to the surface of the sphere. The inverse would be the case for a particle with positive magnetic susceptibility. However, the maximum magnetic field enhancement is in the order of $10^{-5}$ relative to the incident field. Figure 2b shows the gradient of Fig. 2a outside the particle, where the gradient magnetic field reaches the maximum order of $10^4$. This relatively high magnetic field gradient is confined to the immediate vicinity of the sphere.

When considering (2) the polarizability of the particle depends on its radius ${R{{_\text{p}}}}$ and its susceptibility $\chi _{\text{p}}$. Figure 2c shows the secondary magnetic field distribution of the same arrangement as in Fig. 2a along the *x*-axis for different particle sizes. It can be seen that the maximum magnetic field remains the same for all three particle sizes while the maximum gradient of the magnetic field varies significantly with the size of the particle. The maximum magnetic field gradient decreases with increasing particle size.

From (2) it can be deduced that for the spherical particle the magnitude of the polarizability becomes bigger with increasing magnitude $|\chi _{\text{p}}|$. While for ${|\chi _{\text{p}}| \ll 3}$ the polarizability can be approximated with ${\alpha _{\text{m}} = V\chi _{\text{p}}}$, saturation is reached for ${|\chi _{\text{p}}| \gg 3}$ (${\alpha _{\text{m}} = 3V}$). While the quantitative magnetic field distribution changes for different magnetic susceptibilities, the qualitative magnetic field distribution around a spherical particle (location of minima and maxima, and overall field distribution) remains similar to the results shown.

#### Coupling of two spherical particles

Since in biological applications the surface of a nanoparticle is usually modified to improve its biocompatibility, targetability, stability, and functionality^48^, the magnetic susceptibility of the particle can differ significantly from the susceptibility of bulk gold material due to charge carrier redistribution. Diamagnets obtain a magnetic susceptibility smaller than zero, while superconductors are considered *ideal diamagnets* with a magnetic susceptibility of − 1^49,50^. Paramagnetic materials obtain magnetic susceptibilities higher than zero and the magnetic susceptibility of ferromagnetic materials can be in the range of hundreds of thousands as in the case of iron^51^. We choose a wide range of magnetic susceptibilities from ${-0.9\le \chi _{\text{p}} \le 10}$ for our investigations, as to the best of our knowledge, the possible limits of magnetic susceptibilities for surface modified AuNPs are not yet determined^41^. This range is probably wider than realistic magnetic susceptibility values for modified AuNPs; however, it allows us to investigate the limits of possible magnetic field interaction.

In the following, the interaction of two spherical particles as a function of their magnetic susceptibility is analysed.

**Figure 3:**
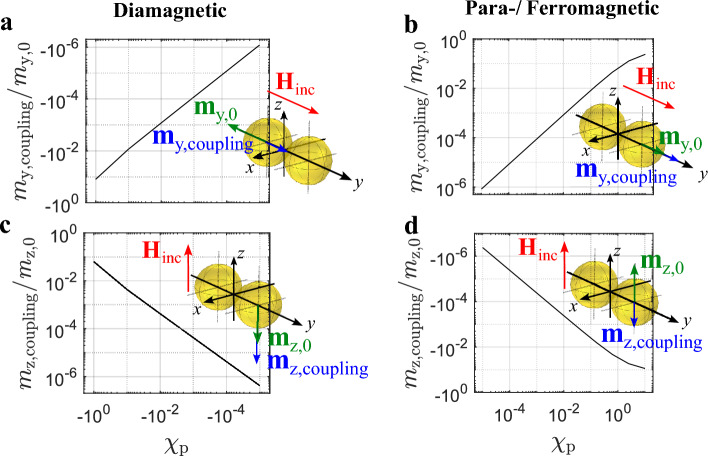
Magnetic dipole moment contribution due to coupling of two identical spherical AuNPs normalized to the magnetic dipole moment of a single particle. (**a**) Diamagnetic particles with incident magnetic field in *y*-direction. (**b**) Para-/ferromagnetic particles with incident magnetic field in *y*-direction. (**c**) Diamagnetic particles with incident magnetic field in *z*-direction. (**d**) Para-/ferromagnetic particles with incident magnetic field in *z*-direction.

Figure 3a–d show two touching AuNPs arranged along the *y*-axis. Furthermore, in Fig. 3a and b, the incident magnetic field is oriented in *y*-direction. Applying the general Eqs. (30) and (31) from the methods section, the magnetic moment of each particle is oriented in *y*-direction and its magnitude can be expressed as

8 $$\begin{aligned} m_{1,\text{y}} = \alpha _{\text{m}}{H}{{_\text{inc,y}}} + \alpha _{\text{m}}{H}{_\text{2,y}}, \end{aligned}$$

while

9 $$\begin{aligned} {H}{{_\text{2,y}}} =\frac{m_{2,\text{y}}}{16\pi {R_{\text{p}}^3}}. \end{aligned}$$

${H}{{_\text{2,y}}}$ is the additional magnetic field contribution from the second particle at the position of the first particle and $m_{2,\text{y}}$ is the magnitude of magnetic dipole moment of the second particle. Since the particles are identical, $m_{1,\text{y}} =m_{2,\text{y}}$. Thus, by considering the polarizability from (2) and reformulation of (8) the following equation can be obtained

10 $$\begin{aligned} m_{1,\text{y}} = \alpha _{\text{m}}{H}{{_\text{inc,y}}}\left( 1 + \frac{\chi _{\text{p}}}{12+3\chi _{\text{p}}}\right) . \end{aligned}$$

Thus, the magnitude of the magnetic dipole moment of each particle from Fig. 3a and b can be calculated with the superposition of the following contributions

11a $$\begin{aligned} m_{\text{y,0}}&= {\mathrm{\alpha _m}}{} \textbf{H}_{\text{inc}}{} \textbf{e}_{\text{y}} , \end{aligned}$$

11b $$\begin{aligned} m_{\text{y,coupling}}&= m_{\text{y,0}} \frac{\chi _{\text{p}}}{12+3\chi _{\text{p}}} . \end{aligned}$$

The index zero refers to the dipole moment of each particle in the absence of interaction with the other particle. The index ’coupling’ indicates the additional magnetic dipole moment resulting from the interaction between the particles.

Considering Fig. 3c and d, the particles are arranged along the *y*-axis and the incident magnetic field is oriented in *z*-direction. By application of the same approach as before, the magnitude of the magnetic dipole moment of each particle from Fig. 3c and d can be calculated with the superposition of the following contributions

12a $$\begin{aligned} m_{\text{z,0}}&= {\mathrm{\alpha _m}}{} \textbf{H}_{\text{inc}}{} \textbf{e}_{\text{z}} ,\end{aligned}$$

12b $$\begin{aligned} m_{\text{z,coupling}}&= -m_{\text{z,0}} \frac{\chi _{\text{p}}}{24+9\chi _{\text{p}}}. \end{aligned}$$

The applicability of the presented formulas was validated by comparison with numerically obtained magnetic dipole moments. The comparison is shown in the Methods section, where it can be seen that the formulas result in very good agreement with numerically obtained magnetic dipole moments.

In Fig. 3a and b the ratio $m_{\text{y,coupling}}/m_{\text{y,0}}$ is plotted and in Figure 3c and d the ratio $m_{\text{z,coupling}}/m_{\text{z,0}}$ is shown. The respective ratios can be positive or negative, depending on the susceptibility $\chi _ {\text{p}}$ and the orientation of the magnetic field vector relative to the particle arrangement. This means that the interaction of the particles can cause an increase or a decrease of magnetic dipole moment. Additionally, in all cases, the coupling increases with increasing magnitude $|\chi _ {\text{p}}|$, while in the case of para- and ferromagnetic particles the onset of saturation is visible around $\chi _ {\text{p}} = 10$.

It should be noted that for higher particle distances the coupling of the particles becomes weaker until the magnetic dipole moment can be approximated with the magnetic dipole moment of individual particles ($\textbf{m} = \alpha _\text{m}{} \textbf{H}{_\text{inc}}$). This is further shown in the “Methods” section.

### Prolate and oblate surface-modified particles

In the following, the interaction of elliptical particles will be treated and compared to the spherical case. Ellipsoidal AuNPs are among the particle shapes commonly used in the frame of biomedicine^20^. Furthermore, it was found that ellipsoidal particles thiolated with polyethylene glycol can exhibit strong diamagnetic properties^37^. We fist concentrate on the magnetic response of individual prolate elliptical particles. We investigate the response as a function of the different axis ratios as well as a function of different magnetic susceptibilities. Subsequently, we show the results for oblate elliptical particles. The investigations of the individual particles allow a target-oriented investigation of the coupling of two particles which are spherical, prolate or oblate elliptical. Since we like to explore the limits of magnetic response, the target is to achieve a maximum in magnetization of the particles. Therefore, we investigate the magnetization as a function of two interacting particles with the different shapes.

#### Prolate elliptical particles

**Figure 4:**
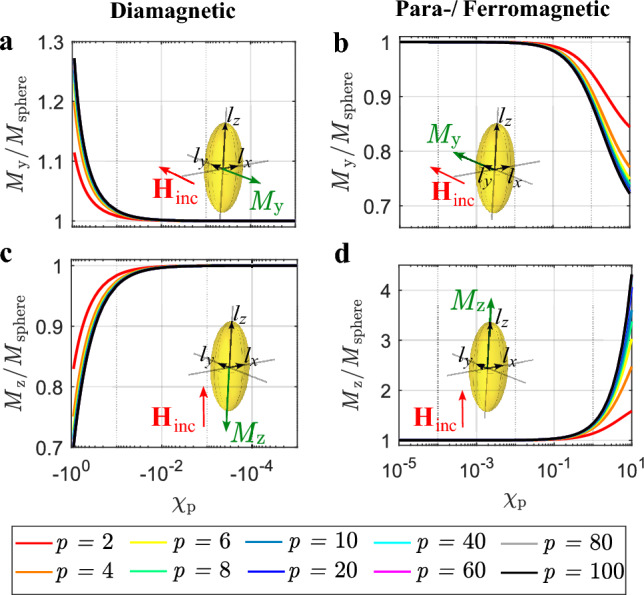
Relative magnetization in a prolate AuNP. (**a**) Diamagnetic particle with incident magnetic field in *y*-direction. (**b**) Para-/ferromagnetic particle with incident magnetic field in *y*-direction. (**c**) Diamagnetic particle with incident magnetic field in *z*-direction. (**d**) Para-/ferromagnetic particle with incident magnetic field in *z*-direction.

Figure 4 depicts prolate elliptical particles with semi-axes of lengths $l_x$, $l_y$ and $l_z$, where $l_z > l_y$ and $l_y = l_x$. The indices denote the orientation of the length parallel to the respective axis, while $l_z/l_y = p$. The parameter *p* equals integer values in the range $2\le p \le 100$.

Figure 4 shows the prolate elliptical particle with the incident magnetic field vector oriented either along the major axis of the particle (Fig. 4c,d) or one of the minor axes (Fig. 4a,b). The magnitude of magnetization $M_i$ of one particle can be calculated with^52^

13 $$\begin{aligned} M_i = \frac{\chi _{\text{p}}}{1+N_i\chi _{\text{p}}}{} \textbf{H}_{\text{inc}}\textbf{e}_{i},\qquad i \in \{x,y,z\}, \end{aligned}$$

where $N_i$ is a shape dependent (demagnetization) factor, since the shape of the particle affects the magnetic field distribution. The Cartesian axes are elements of the index *i* and indicate the orientation of the magnetization. Since a sphere has point-symmetry relative to its center, its demagnetization factor is constant in all directions and equals $N_i = 1/3$ ^53^. The formulas (35) and (36) in the “Methods” section allow the calculation of the demagnetization factors for prolate elliptical particles.

The graphs in Fig. 4 show the relationship between the respective magnetization of the particle relative to the magnetization of a sphere, as a function of the particle’s susceptibility, and different ratios of the major axis length to the minor axis length ($l_z/l_y = p$). Depending on the magnetic susceptibility and orientation of the magnetic field vector relative to the particle, the magnetization of the prolate particle can be greater or lower than the magnetization of a sphere with the same susceptibility. The biggest enhancement in magnetization is found for $\chi _ {\text{p}} > 1$ with the magnetic field vector oriented parallel to the particle’s major axis (Fig. 4d).

#### Oblate elliptical particles

**Figure 5:**
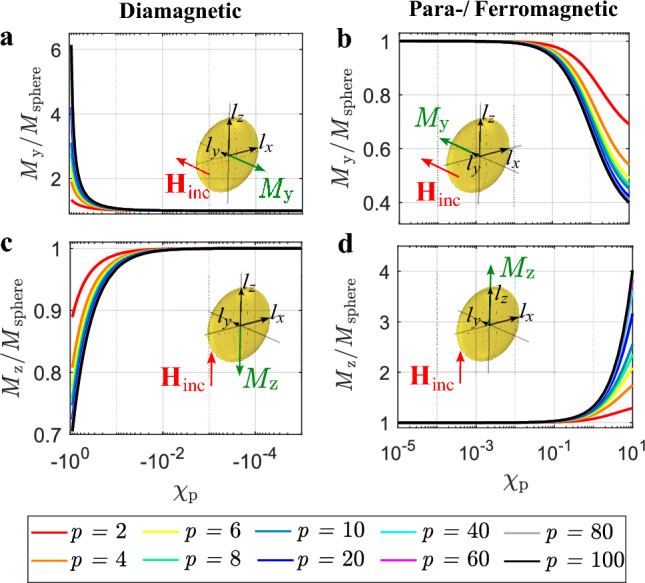
Relative magnetization in an oblate AuNP. (**a**) Diamagnetic particle with incident magnetic field in *y*-direction. (**b**) Para-/ferromagnetic particle with incident magnetic field in *y*-direction. (**c**) Diamagnetic particle with incident magnetic field in *z*-direction. (**d**) Para-/ferromagnetic particle with incident magnetic field in *z*-direction.

Similarly to the results from Fig. 4, the graphs in Fig. 5 show the magnetization of oblate particles relative to the magnetization of a sphere with the same magnetic susceptibility. The magnetization of the oblate elliptical particle is calculated with (13) and by application of the formulas (37) and (38) for the demagnetization factor. The highest magnetization occurs when the incident magnetic field vector is perpendicular to the minor axis of the oblate particle and $\chi _ {\text{p}} > 1$ (Fig. 5d).

When comparing Fig. 5d to 4d, it is evident that the prolate particle induces a much higher magnetization at $\chi _ {\text{p}} > 1$ than the oblate particle. Interestingly, when comparing the diamagnetic particles in Fig. 5a and 4a, the oblate particle yields a higher magnetization.

#### Coupling of the differently shaped particles

The comparison of Figs. 4 and 5 allows assumptions of the magnetic field interaction of the individual particles, especially the quantification of the impact of the three different shapes (spherical, prolate and oblate ellipsoidal). However, the interaction of elliptical particles is yet to be investigated and compared to the interaction of spherical particles.

**Figure 6:**
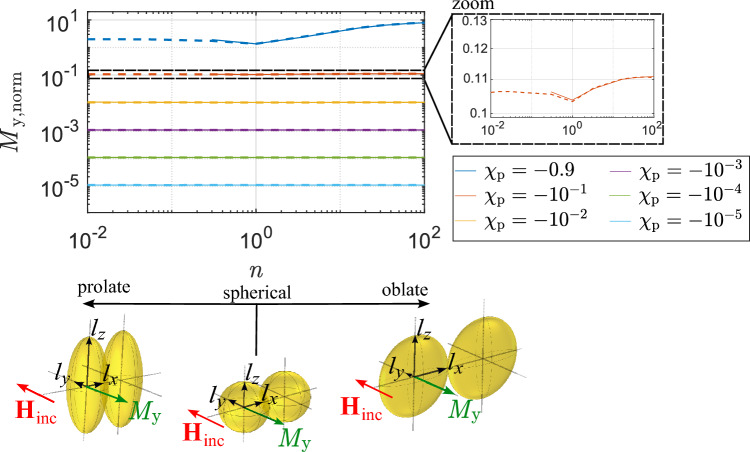
Normalized magnetization of diamagnetic particles with different shapes and axis ratios. Solid lines are results obtained with presented analytical formulas. Dashed lines are results obtained with numerical EM simulations.

In this subsection, we investigate the limits of magnetic responses and search for a configuration of two identical particles that results in maximum magnetization. First, we consider diamagnetic particles. Figure 3c shows the configuration that generates the highest magnetic dipole moment for diamagnetic spherical particles. Positive coupling of diamagnetic particles can be achieved when the incident magnetic field is perpendicular to the line connecting the particle centers (e.g. the incident magnetic field in *z*-direction and the arrangement of particles in *y*-direction as in Fig. 3c). Additionally, for elliptical diamagnetic particles, magnetization is highest when the magnetic field is oriented along one of the semi axes (as seen in Figs. 4a and 5a). The maximum coupling of particles is achieved when they are brought as closely together as possible. Based on these information, Fig. 6 shows the configurations of diamagnetic particles with different shapes that result in maximum magnetization. The graph illustrates $M_{\text{y,norm}}$, which is the magnitude of magnetization in *y*-direction, normalized to the incident magnetic field and can be expressed by application of the magnetic dipole moment:

14 $$\begin{aligned} M_{\text{y,norm}}=\frac{m_{\text{y}}}{V{H}{{_\text{inc,y}}}}. \end{aligned}$$

Note that in this definition the magnetization is normalized to the incident magnetic field to avoid the dependence of the parameter from the incident field. When approximating the particles as point dipoles, the general Eqs. (30) and (31) from the methods section can be applied to calculate the dipole moments. This results in a similar approach as in the case of the coupling of two spheres and the magnetic dipole moments of the particles from Fig. 6 can be expressed with

15 $$\begin{aligned} m_{\text{y}} = \alpha _{\text{m,y}}{H}{{_\text{inc,y}}} - \alpha _{\text{m,y}}\frac{m_{\text{y}}}{32\pi l_{\text{x}}^3}. \end{aligned}$$

$\alpha _{\text{m,y}}={\chi _{\text{p}}V}/({1+N_{\text{y}}\chi _{\text{p}}})$ is the polarizability of the particle in *y*-direction. Solving (15) for $m_{\text{y}}$ and inserting the result into (14), the following equation can be obtained

16 $$\begin{aligned} M_{\text{y,norm}}=\frac{\alpha _{\text{m,y}}}{V}\left( 1 - \frac{\alpha _{\text{m,y}}}{32\pi l_{\text{x}}^3+\alpha _{\text{m,y}}}\right) . \end{aligned}$$

In Fig. 6, the different colors represent the different susceptibilities. $M_{\text{y,norm}}$ is plotted as a function of the configurational parameter *n*, which represents the ratio of the lengths of the axes

17 $$\begin{aligned} n={\left\{ \begin{array}{ll} 1/p &{} \text { For  \,  prolate  \,  particles} \\ p &{} \text { For \,  oblate \,  particles} \end{array}\right. } . \end{aligned}$$

The numerically obtained results from Comsol Multiphysics are depicted using dashed lines, while the results obtained analytically with (16) are represented with solid lines. Generally, the numerically and analytically obtained results are in good agreement, with the magnetization increasing with higher magnetic susceptibility magnitudes, which is unsurprising. It can be noticed, that for $\chi _ {\text{p}} \le -10^{-1}$ the analytical results are obtained for $n>0.3$. The reason thereof is that a limit for the applicability of (31) is reached, which is used for the derivation of (16). For a very long prolate particle’s short-range area, the point dipole approximation is not valid. However, for all other susceptibility values and shapes, there is significant agreement between the analytical and numerical results.

At $\chi _ {\text{p}} = -0.9$, it is noticeable that the oblate particle with the maximum ratio of major axis to minor axis (*n*) generates the highest magnetization. This trend can also be observed in magnified inset on the other curves. Additionally, in magnified inset, it is observed that both prolate and oblate particles yield higher magnetization than spherical particles.

**Figure 7:**
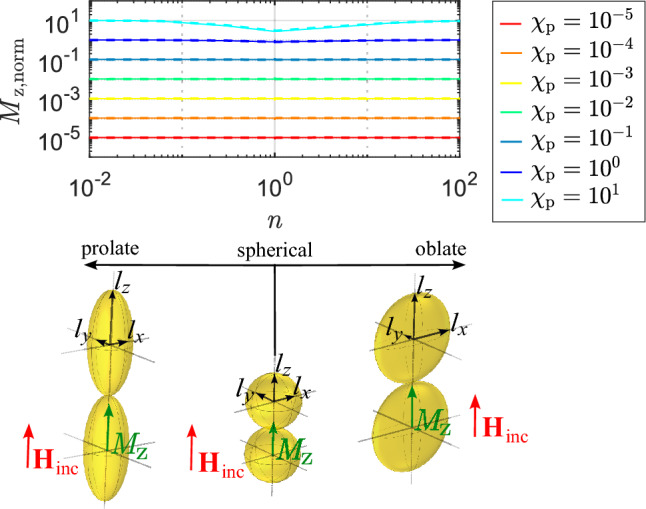
Normalized magnetization of para-/ ferromagnetic particles with different shapes and axis ratios. Solid lines are results obtained with presented analytical formulas. Dashed lines are results obtained with numerical EM simulations.

Figure 7 illustrates a result similar to that shown in Fig. 6, but for the coupling of para-/ferromagnetic particles. The principle mathematical approach is similar to the derivation of (16) and the normalized magnetization is

18 $$\begin{aligned} M_{\text{z,norm}}=\frac{m_{\text{z}}}{V{H}{{_\text{inc,z}}}} = \frac{\alpha _{\text{m,z}}}{V}\left( 1 + \frac{\alpha _{\text{m,z}}}{16\pi l_{\text{z}}^3-\alpha _{\text{m,z}}}\right) . \end{aligned}$$

Figure 7 demonstrates excellent agreement between the numerically and analytically obtained results. The prolate particles exhibit the highest magnetization which is in contrast to the diamagnetic particles, where the oblate particles yield to highest magnetization.

### Example: spherical arrays of particles

**Figure 8:**
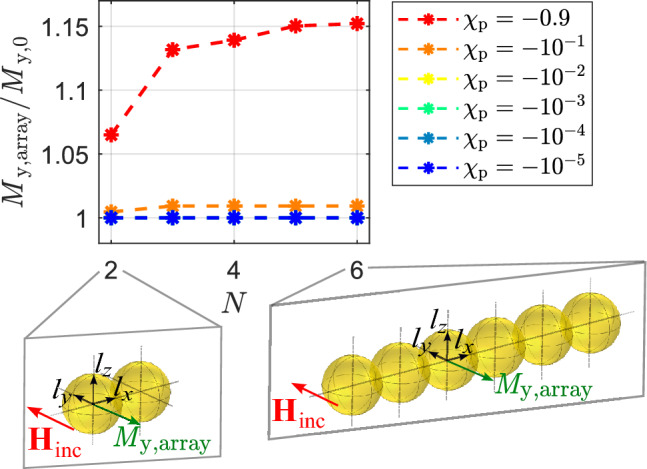
Maximum magnetization in a line of spherical diamagnetic particles normalized to the magnetization of a single particle without in absence of the other particles.

As already mentioned in the introduction one major study used surface modified AuNPs for biological reprogramming^28^. There, fibroblast cells were planted on an array of spherical particles and exposed to a magnetic field^28^. The arrangement of particles raises the question: how do the particles interact with each other? Specifically, we seek to determine the number of particles that interact with one another in such an array. To determine the number of particles that interact with each other in an array, we first focus on a line of particles, as illustrated in Fig. 8. To enhance the magnetization of the diamagnetic particles in the array due to their interactions, as in the result of Fig. 3c, we applied a magnetic field perpendicular to the direct connection vectors between the particle centers. We gradually increased the number of particles in a line from $N=2$ to $N=6$, as shown in Fig. 8. The figure shows the ratio of the maximum magnetization ($M_{\text{y,array}}$) within the line of particles to the magnetization of a single particle without any interactions ($M_{\text{y,0}}$). The asterisks indicate the corresponding $M_{\text{y,array}}/M_{\text{y,0}}$ values for each particle number, while the dashed lines show how this ratio changes with *N*. The colors in the figure represent different magnetic susceptibility values. The susceptibility curves for $\chi _{\text{p}} \ge -10^{-2}$ overlap, indicating a lack of significant increase in magnetization due to particle interaction at these levels. However, for susceptibility values lower than $\chi _{\text{p}} \le -10^{-1}$, there is a relevant increase in $M_{\text{y,array}}/M_{\text{y,0}}$, showcasing meaningful particle interaction. The maximum interaction between particles saturates at $N = 3$ for $\chi _{\text{p}} = -10^{-1}$, whilst for $\chi _{\text{p}} = -0.9$, saturation is reached at $N = 5$ particles. Based on the susceptibility values considered, it can be inferred that a maximum of five particles aligned in a single row can result in relevant enhancement in magnetization. Expanding the number of particles in the row only leads to an increase in the area covered by the maximum magnetization, but does not enhance the maximum magnetization itself.

**Figure 9:**
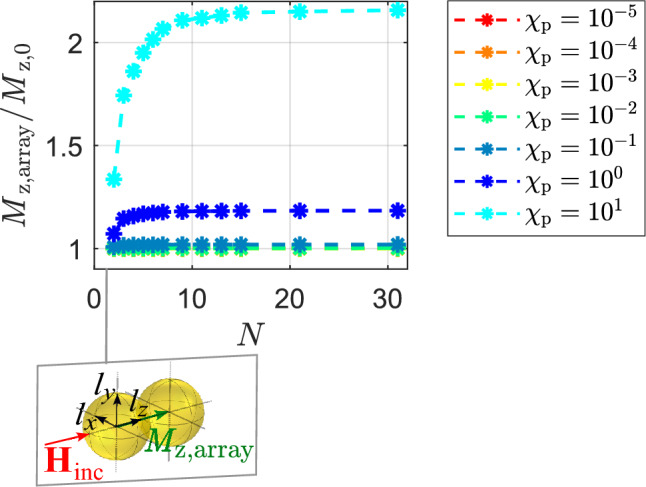
Maximum magnetization in a line of spherical para-/ ferromagnetic particles normalized to the magnetization of a single particle without in absence of the other particles.

Similar to Figs. 8 and 9 shows results for particle interaction in the form of $M_{\text{y,array}}/M_{\text{y,0}}$ for para-/ ferromagnetic particles. The arrangement of particles relative to the incident magnetic field is chosen on the basis of the results from Fig. 3a to obtain an enhancement of magnetization. The results show that the onset of increased $M_{\text{y,array}}/M_{\text{y,0}}$ begins at $\chi _{\text{p}} \ge 10^{-1}$, and the number of interacting particles increases with higher magnetic susceptibility values. Saturation is reached at $N = 9$ for $\chi _{\text{p}} = 10$.

**Figure 10:**
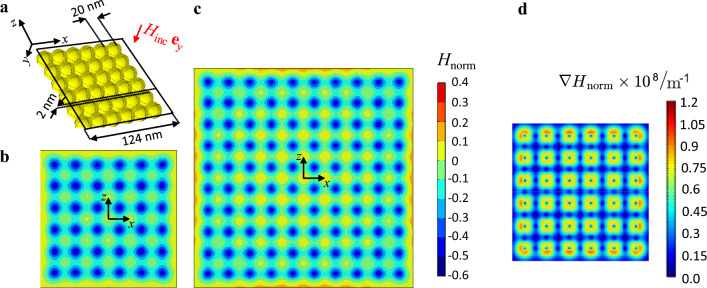
Array of spherical diamagnetic particles. (**a**) Schematic illustration of the $6\times 6$ particles array. (**b**) Secondary magnetic field distribution above the array. (**c**) Secondary magnetic field distribution above a $10\times 10$ particles array. (**d**) Gradient magnetic field of the result from (**b**).

Returning to an array of diamagnetic particles as used in a study for biological reprogramming of cells^28^, we will now investigate the limits of magnetic field alteration due to the particles interaction. Figure 10a shows an array of AuNPs, six particles wide and six particles tall, exposed to a magnetic field perpendicular to the array plane. The particles have a diameter of ${R_{\mathrm{{p}}} = 10\,\text{nm}}$ and are placed directly beneath each other without any additional spacing. The magnetic field $H_{\text{norm}}$ is determined within the black rectangular area displayed in Fig. 10a, which is then illustrated in Fig. 10b. The same arrangement is analyzed for an array of 10 by 10 nanoparticles, as depicted in Fig. 10c. Note that Fig. 10b and c use the same scale. Comparing the two plots, it is evident that there is no significant enhancement in the magnetic field due to more particles. Although the alignment of the particles enables an enhancement in magnetization, it leads to an increase in magnetic field between the particles and a reduction in magnetic field on the particles’ surface. This results in a range of $-0.5 \le H_{\text{norm}} \le 0$, showing that the magnetic field above the array is lower than the incoming field.

The magnetic field gradient of the arrangement from Fig. 10a and b is shown in Fig. 10d. The magnetic field gradient peaks at approximately $10^8\,\mathrm{m^{-1}}$.

**Figure 11:**
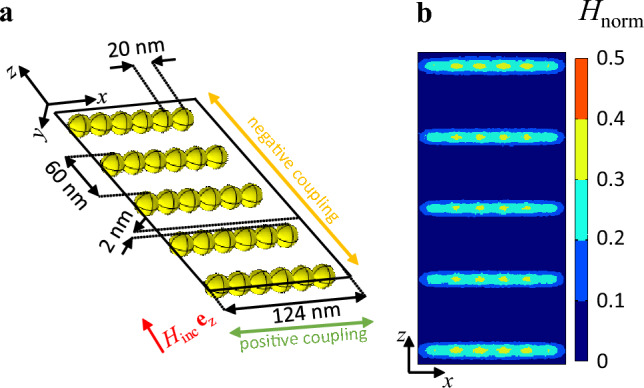
Modified array of spherical diamagnetic particles. (**a**) Schematic illustration. (**b**) Secondary magnetic field distribution above the array.

The magnetic field distribution from Fig. 2a shows that the secondary magnetic field around an AuNP is maximum at the positions where the incident magnetic field vector is only tangential to the surface of the particle. To obtain a magnetic field enhancement above an array of diamagnetic particles, this characteristic is exploited. In contrast to the arrangement from Fig. 10a and 11a shows a configuration where a partial enhancement of the magnetic field on top of the particles is excited. The incoming magnetic field is running parallel to the *z*-direction. The particles are closely positioned in the *x*-direction while there is a 60 nm distance between neighboring particles in the *z*-direction. The spacing results in a decrease of negative coupling (as in the result of Fig. 3a) between the diamagnetic particles. By implementing this configuration, the magnetic field is increased by a factor of 0.3 directly above the particles.

The findings from Fig. 6 indicate that the magnetization and magnetic field interaction of particles could be enhanced by utilizing prolate/oblate particles with low/ high configurational parameter *n*. However, when dealing with spherical diamagnetic particles, achieving magnetic field enhancement in the range of the incident magnetic field necessitates magnetic susceptibility values in the range of $\chi _ {\text{p}} = -0.9$ and a very specific particle arrangement, as chosen in Fig. 11.

## Discussion

We presented analytical formulas for the calculation of the magnetic response of individual spherical particles for frequencies up to several hundreds of Megahertz. These formulas provided the basis of our following investigations. All our analytically obtained results are validated with numerical electromagnetic simulations in Comsol Multiphysics.

We showed that the magnetization of an AuNP is dominated by the polarizability $\alpha _{\text{m}}$ as long as the magnetic field excitation fulfills $f < 100\,\text{MHz}$ and $R_{\text{p}} \le 100\,\text{nm}$. We showed the magnetic field distribution within and around an AuNP with bulk gold material properties, as well as its magnetic field gradient. These results showed that the magnetic field around an AuNP is partially enhanced and partially decreased, depending on the considered position. However, the alteration in the magnetic field is approximately five orders of magnitude smaller than the incident magnetic field while the maximum magnetic field gradient is in the order of $10^5$. Considering that the magnetic energy *E* of a particle is ^54^

19 $$\begin{aligned} E = -\frac{\mu _0\chi _{\text{p}}V}{2}H^2, \end{aligned}$$

($\mu _0$ is the vacuum permeability) the magnetic field contribution of an AuNP with bulk gold material parameters is too small to cause significant energy alterations. Furthermore, the magnetic force per particle volume $\textbf{f}$ is^54,55^

20 $$\begin{aligned} \textbf{f} = {\mu _0\chi _{\text{p}}}(\textbf{H}\cdot \nabla )\textbf{H}. \end{aligned}$$

Thus, to achieve a magnetic force density in the range of gravitational force density which is approximately $f_{\text{g}}=10^4 \mathrm{N/m^3}$ ^55^, very high magnetic fields in combination with magnetic field gradients are necessary. However, we also showed that the gradient of the magnetic field can be tuned with the particle size.

Furthermore, the magnetic dipole moments of two neighboring spherical particles in contact can be enhanced or lowered due to their interaction. In this regard, the determining factors are orientation of the magnetic field relative to the arrangement of nanoparticles, and the susceptibility of the particles.

Since the magnetization of a particle is proportional to its magnetic field response, we investigated the magnetization of prolate and oblate ellipsoids relative to the magnetization of a spherical particle. We considered different axis ratios for the elliptical shapes and also accounted for different magnetic susceptibilities. Thereby, we showed that the maximum magnetization (hence maximum magnetic field) can be reached for prolate para-/ferromagnetic ellipsoids and oblate diamagnetic ellipsoids. Furthermore, depending on the orientation of the magnetic field relative to the elliptical particle, the magnetization can also be lower than the magnetization of spheres.

Subsequently, we investigated and compared the magnetic coupling of two particles with the different shapes. Here, we saw that in the cases of positive interaction (interaction causing an increase in magnetization), the elliptical particles show stronger interaction than spherical particles. We showed that for diamagnetic particles, the oblate ellipsoids show the strongest interaction while for para-/ferromagnetic particles the prolate ellipsoids interact most strongly.

Emanating from an example taken from literature, we investigated the interaction of spherical particles in an array. We found that the determining coupling factor is the magnitude of the magnetic susceptibility: the higher the magnitude $|\chi _{\text{p}}|$, the more particles can interact with each other. However, for the considered susceptibility range ${-0.9\le \chi _{\text{p}} \le 10}$ in case of diamagnetic particles maximum five particles can couple effectively in one row and in case of ferromagnetic particles, maximum fifteen particles can couple effectively in one row. Finally, we exemplary investigated the magnetic field and the magnetic field gradient above arrays of diamagnetic nanoparticles. The main findings of these investigations are that for the case of positive coupling of particles, depending on the orientation of the incident magnetic field relative to the array surface, the incident magnetic field can be enhanced or lowered. Our results show that an extreme diamagnetic susceptibility of $\chi _{\text{p}}=-0.9$ can achieve a magnetic field enhancement in the range of the incident magnetic field. Since there is the hypothesis that a relevant increase in magnetic field was reached with strong diamagnetic AuNPs experimentally^28^, we determine that the magnetic susceptibility for such an increase in the magnetic field must be in the range of ${\chi _{\text{p}}}=-0.9$. As mentioned before, the limit of possible susceptibility values for surface-modified AuNPs is yet to be determined. However, a value in the range of ${\chi _{\text{p}}}=-0.9$ is beyond realistic susceptibility values found in literature thus far (see Introduction).

We would like to emphasize that to the best of our knowledge, the exact physics of the magnetism in functionalized AuNPs is not finally understood. Our study provides researchers with a methodology to estimate the magnetic response of functionalized AuNPs with known magnetic susceptibility. We identified formulas which are applicable, and showed their validity with numerical electromagnetic simulations. In contrast to numerical methods, analytical approaches directly show the connection between different parameters (as shape, size and number of particles) and the magnetic response. The understanding of the low-frequency magnetic response of AuNPs is essential for further determination of the interaction of the magnetic field energy with biological cells and cell compartments. Our findings contribute to a better understanding of the limits, characteristics, and impact of AuNPs on the magnetic field response, with potential applications in biomedical research and treatment. Therefore, our investigation provides important guidance and contributes to a better understanding of the low-frequency magnetic response of AuNPs.

However, we believe that further research is needed to comprehend the physics behind the enhanced reprogramming efficiency observed^28^ when using functionalized AuNPs in combination with magnetic fields. The original hypothesis that an enhanced magnetic field due to AuNPs causes a higher reprogramming efficiency seems unlikely, because for realistic magnetic susceptibility values the enhancement of the magnetic field is orders below the incident magnetic field. Since the exact physics behind functionalized AuNPs are not known yet, a further direction to be investigated is the fundamental investigations on the properties of materials, especially the exact relation between factors that affect the magnetic susceptibility and the magnetic susceptibility itself. Furthermore, the relevance of non-uniform magnetic field distributions for the interaction of AuNPs and cells needs to be investigated since AuNPs cause relatively high magnetic field gradients in their close vicinity. Finally, to understand the interaction of magnetic fields with surface-modified AuNPs and cells, different modelling principles considering multiphysics can be applied, e.g. molecular dynamics or density functional theory.

## Methods

### Numerical electromagnetic simulations

Numerical simulations were performed using the Magnetic Fields (mf) interface under the AC/DC Electromagnetic Fields branch in Comsol Multiphysics (COMSOL AB, Sweden, https://www.comsol.com). Due to high water content, the effective magnetic susceptibility of cells, including major compartments like the cytosol, is in the range of ${10^{-6}}$^55,56^. Lipid structures in cells have even lower magnetic susceptibilities than compartments with high water content ^55^. However, due to their higher magnetic susceptibility, the magnetic response of AuNPs to low-frequency magnetic fields is generally much stronger than the response of eukaryotic cells. Therefore, in our investigations, we neglect the specific cellular environment and consider AuNPs located in air. The geometry consisted of the gold nanoparticles with the corresponding shapes and material properties for the respective investigation, located inside an (spherical) environment with vacuum material properties. An additional layer around the environment built up the infinite element domain to absorb outgoing EMFs. The incident magnetic field was excited by setting a background magnetic vector potential which resulted in the target incident magnetic field. However, when evaluating the results, the total magnetic field was normalized to the incident magnetic field. The target frequencies of the respective investigations were set in the study section and material sweeps were conducted where necessary.

Magnetic dipole moments were determined numerically in the derived values section, by volume integration of the magnetization.

### Fundamental analytical calculations

#### Ampère’s circuital law and the magnetic polarization definition

Ampère’s circuital law in differential form and in frequency domain describes the rotation of the magnetic field $\textbf{H}$ as the sum of different current density contributions,

21 $$\begin{aligned} \nabla \times {\textbf{H}}=(\sigma -\text {i}\omega \varepsilon ){\textbf{E}}+\nabla \times {\textbf{M}}\equiv \textbf{j}_\text{e}+\textbf{j}_\text{m} . \end{aligned}$$

In Eq. (21) $\textbf{E}$ is the electric field vector, $\sigma$ is the electrical conductivity and $\varepsilon =\varepsilon _0\varepsilon _{p,r}$ is the material permittivity, while $\textbf{M}$ is the magnetization of it. The first summand in (21) describes the conductive and displacement current density $\textbf{j}_\text{e}$ and the second summand describes a current density $\textbf{j}_\text{m}$ due to magnetization. The magnetic dipole moment $\textbf{m}$ of the considered volume can be expressed with a volume integral over the current densities from (21),

22 $$\begin{aligned} \begin{aligned} \textbf{m}&=\frac{1}{2} \iiint \limits _{{V}}^{}{\textbf{r}}\times ((\sigma -\text{i}\omega \varepsilon ){\textbf{E}}+\nabla \times {\textbf{M}}) {\text{d}}{V} =\frac{1}{2} \iiint \limits _{{V}}^{}{\textbf{r}}\times (\sigma -\text{i}\omega \varepsilon ){\textbf{E}} {\text{d}}{V} +\frac{1}{2} \iiint \limits _{{V}}^{}{\textbf{r}}\times \nabla \times {\textbf{M}} {\text{d}}{V} \\&= \frac{1}{2} \iiint \limits _{{V}}^{}{\textbf{r}}\times {\textbf{j}}_\text{e} {\text{d}}{V} +\frac{1}{2} \iiint \limits _{{V}}^{}{\textbf{r}}\times {\textbf{j}}_\text{m} {\text{d}}{V}= \textbf{m}_\text{e}+\textbf{m}_\text{m}\\&= \alpha _\text{e}{\textbf{H}}{_\text{inc}}+\alpha _\text{m}{\textbf{H}}{_\text{inc}} . \end{aligned} \end{aligned}$$

*V* is the volume of the particle, and $\textbf{r}$ is the position vector. Furthermore, the magnetic dipole moment is generally defined as the product of a magnetic polarizabilitiy and the incident magnetic field. We define $\alpha _\text{m}$ as the magnetic polarizability which is connected to the magnetization $\textbf{M}$ and $\alpha _\text{e}$ as the magnetic polarizability which is connected to the conductive and displacement currents.

In order to obtain expressions for polarizabilities, it is convenient to consider separately problems with different current sources. In the case of $\textbf{j}_\text{m}=\nabla \times {\textbf{M}}$, we can make the following transformations^44,45^

23 $$\begin{aligned} \textbf{m}_\text{m}=\frac{1}{2} \iiint \limits _{{V}}^{}{\textbf{r}}\times \nabla \times {\textbf{M}} {\text{d}}{V}=\iiint \limits _{{V}}^{}{\textbf{M}} {\text{d}}{V}=3\iiint \limits _{{V}}^{}\frac{\mu _r-1}{\mu _r+2}\textbf{H}_\text{inc} {\text{d}}{V}=3{V}\frac{\mu _r-1}{\mu _r+2}{} \textbf{H}_\text{inc}\,, \end{aligned}$$

where, $V=4\pi R^3/3$ (*R* is the particle radius), and we used the solution to the static problem of a magnetic sphere with relative magnetic permeability $\mu _r$ in a constant external magnetic field $\textbf{H}_\text{inc}$^45^. Note that this solution can be used because we assume from the very beginning that the particle is much smaller than the wavelength of the external field. Thus, we can write in this approximation that

24 $$\begin{aligned} \alpha _\text{m}=3{V}\frac{\mu _r-1}{\mu _r+2}\,. \end{aligned}$$

In general the magnetic dipole moment contribution due to magnetization $\textbf{M}$ has a static contribution and a frequency-dependent contribution^44^. However, in the considered frequency regime from Fig. 1, the static contribution is dominant leading to the static magnetic polarizability $\alpha _\text{m}$ from (2).

The case of $\textbf{j}_\text{e}=(\sigma -\text{i}\omega \varepsilon ){\textbf{E}}$ is more complicate and can not be considered in the static approximation because we must take into account the connection between electric and magnetic fields corresponding to the Maxwell equation:

25 $$\begin{aligned} \nabla \times {\textbf{E}}=\text{i}\omega \mu _0\textbf{H}\,. \end{aligned}$$

Applying the operator $\nabla \times$ to (21) and accounting only $\textbf{j}_\text{e}$ and (25) we obtain

26 $$\begin{aligned} \nabla \times \nabla \times {\textbf{H}}=(\sigma -\text{i}\omega \varepsilon ) \nabla \times {\textbf{E}}=(\sigma -\text{i}\omega \varepsilon )\text{i}\omega \mu _0\textbf{H}=k^2\textbf{H}, \end{aligned}$$

where $k=\omega \sqrt{\varepsilon _0\mu _0\varepsilon _p}$, and $\varepsilon _p=\varepsilon _{p,r}+\text{i}\sigma /(\omega \varepsilon _0)$. Finally, for the magnetic field inside and outside the particle we have the equations

27 $$\begin{aligned} \Delta \textbf{H}+k^2\textbf{H}=0\,\quad \mathrm{(inside)}, \qquad \nabla \times \textbf{H}=0,\,\, \text{and}\,\, \nabla \cdot \textbf{H}=0 \,\quad \mathrm{(outside)} \end{aligned}$$

where to write the equation for the inside we used that $\nabla \times \nabla \times =-\Delta +\nabla (\nabla \cdot )$ and $\nabla \cdot \textbf{H}=0$. The solution of (27) for a spherical particle has been considered elsewhere^47^, where it is shown that the total magnetic field outside a particle can be presented as a superposition of the incident external field and the field generated by the particle’s magnetic dipole moment $\textbf{m}_\text{e}$ determined by the polarizability

28 $$\begin{aligned} \alpha _\text{e}=-\frac{3}{2}V\left( 1-\frac{3}{R^2k^2}+\frac{3}{Rk}\text{cot}\,(Rk)\right) \,. \end{aligned}$$

If the particle size is so small that $|Rk|\ll 1$ then

29 $$\begin{aligned} \alpha _\text{e}\approx \frac{VR^2k^2}{10}\,. \end{aligned}$$

This coincides with (3).

#### Main formulas for interacting particles in the magnetic dipole coupling model

The magnetic dipole moment of one individual particle is excited by the incident magnetic field. When other particles are added, they change the initial magnetic field which is experienced by the first particle and influence its magnetic dipole moment. In a configuration of *N* identical particles, where ${{ N} \in \mathbb {N}, { N} \ge 2}$, the magnetic moment of each particle can be calculated with

30 $$\begin{aligned} \textbf{m}{_ {\text{n}}} ={\mathrm{\alpha _m}} (\textbf{H}_{\text{inc}} + \sum _{k=1, k\ne n}^{N} \textbf{H}_{\text{k}}(\textbf{r}_{\text{n}}-\textbf{r}_{\text{k}})). \end{aligned}$$

The index n indicates the n-th particle and the index k indicates the other particles which interact with it. The magnetic field contribution of the k-th particle can be calculated with ^57^

31 $$\begin{aligned} \textbf{H}_{\text{k}} = \frac{1}{4\pi }(-\frac{\textbf{m}{_ {\text{k}}}}{r^3} + \frac{3(\textbf{m}{_ {\text{k}}}\cdot \textbf{r})\textbf{r}}{r^5}), \end{aligned}$$

where $$r = |\textbf{r}|$$.

#### Numerical validation of the formulas for the magnetic dipole moment of two touching spherical particles

**Figure 12:**
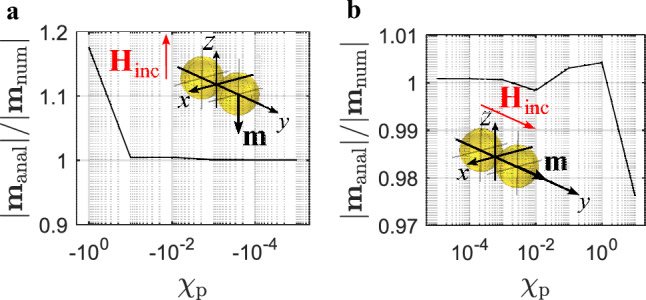
Ratio of analytically obtained magnetic dipole moment magnitude $|\mathbf {m{{_\text{anal}}}}|$ to numerically obtained magnetic dipole moment magnitude $|\mathbf {m{{_\text{num}}}}|$ as a function of magnetic susceptibility. (**a**) Interaction of particles according to Fig. 3c and (**b**) interaction of particles according to Fig. 3b.

Figure 12a shows two touching diamagnetic particles which are arranged along the *y*-axis and which are exposed to an incident magnetic field parallel to the *z*-axis. This configuration equals the arrangement of the particles from Fig. 3c, which resulted in positive interaction of particles. Due to symmetry, the magnetic dipole moment vector $\textbf{m}$ of both particles is equal in magnitude and is directed in negative *z*-direction. To validate the analytical formulas derived for the determination of the magnetic dipole moment, the analytically obtained magnetic dipole moment $|\mathbf {m{{_\text{anal}}}}|$ was compared to the numerically obtained magnitude $|\mathbf {m{{_\text{num}}}}|$. In Fig. 12a the ratio $|\mathbf {m{{_\text{anal}}}}|/|\mathbf {m{{_\text{num}}}}|$ can be seen as a function of different diamagnetic susceptibilities. For susceptibility values down to $\chi _{\text{p}}\thickapprox -10^{-1}$ the graph shows that the analytical result and the numerical result are in excellent agreement ($|\mathbf {m{{_\text{anal}}}}|/|\mathbf {m{{_\text{num}}}}|\thickapprox 1$). For $\chi _{\text{p}}<-10^{-1}$ the ratio increases until it reaches $|\mathbf {m{{_\text{anal}}}}|/|\mathbf {m{{_\text{num}}}}|\thickapprox 1.18$ for $\chi _{\text{p}}\thickapprox -1$. Figure 12b shows a similar graph but for two para-/ ferromagnetic particles in the same arrangement as shown in Fig. 3b. Figure 12b shows that for susceptibilities up to $\chi _{\text{p}}\thickapprox 1$, the analytically and numerically obtained results are in very good agreement ($|\mathbf {m{{_\text{anal}}}}|/|\mathbf {m{{_\text{num}}}}|\thickapprox 1$). For $\chi _{\text{p}} > 1$ the ratio decreases until $|\mathbf {m{{_\text{anal}}}}|/|\mathbf {m{{_\text{num}}}}|\thickapprox 0.98$. The two graphs from Fig. 12 show that in general there is good agreement between the analytically obtained magnetic dipole moment and the numerically obtained magnetic moment, which speaks in favor for the presented analytical formulas.

**Figure 13:**
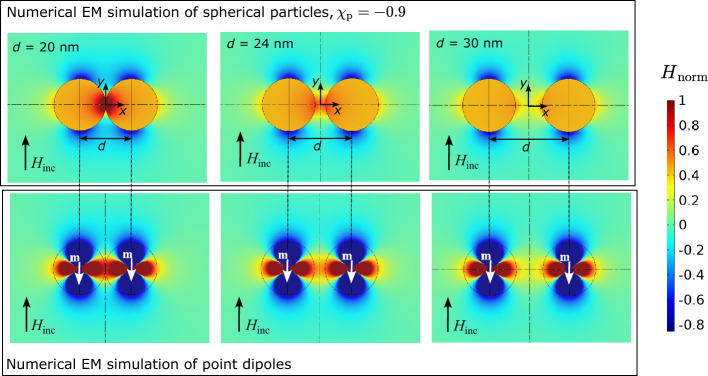
Numerical EM simulation of spherical particles at different distances *d* and comparison with numerical simulation of magnetic point dipoles with the same dipole moment $\textbf{m}$ as the spherical particles at the same distance. View on the longitudinal axis cross-section of the sphere through the center ($z=0$).

Furthermore, Fig. 13 shows the numerical EM simulation of diamagnetic spherical particles with $\chi _{\text{p}} = -0.9$ at different distances *d*. Additionally, Fig. 13 shows the numerical EM simulation results of point dipoles approximating the spherical particles at respective distances with a magnetic dipole moment $\textbf{m}$. All plots from Fig. 13 show $H_{\text{norm}}$ in the same scale. The magnetic field distributions resulting from the point dipoles approximation and the respective magnetic field distributions resulting from the spherical particles are in very good agreement when considering the regions outside the sphere. In the arrangements of particles from Fig. 13 the maximum magnetic field enhancement can be obtained when the particles are brought as closely together as possible ($d = 20\,{\text{nm}}$). When the distance between the particles is increased, the magnetic field enhancement becomes smaller until it equals the maximum magnetic field enhancement of individual particles. This can also be shown analytically. Considering (30) with (31) the magnetization $\textbf{m}$ of one point dipole from Fig. 13 can be expressed with

32 $$\begin{aligned} \textbf{m} ={\mathrm{\alpha _m}} \textbf{H}_{\text{inc}} - \frac{{\mathrm{\alpha _m}}}{4\pi d^3}{} \textbf{m} = -m \textbf{e}_{\text{y}}={\mathrm{\alpha _m}} { H}_{\text{inc}} \textbf{e}_{\text{y}}+ \frac{{\mathrm{\alpha _m}}}{4\pi d^3}m \textbf{e}_{\text{y}} . \end{aligned}$$

With Eq. (32), the magnitude *m* can be obtained as

33 $$\begin{aligned} \textbf{m} ={\mathrm{\alpha _m}} \textbf{H}_{\text{inc}} - \frac{{\mathrm{\alpha _m}}}{4\pi d^3}{} \textbf{m} = -m \textbf{e}_{\text{y}}={\mathrm{\alpha _m}} { H}_{\text{inc}} \textbf{e}_{\text{y}}+ \frac{{\mathrm{\alpha _m}}}{4\pi d^3}m \textbf{e}_{\text{y}} . \end{aligned}$$

**Figure 14:**
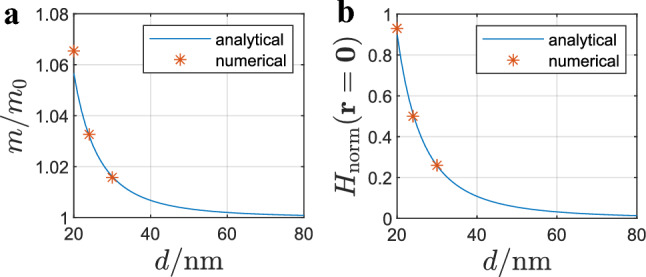
Numerical EM simulation of spherical particles at different distances *d* and comparison with numerical simulation of magnetic point dipoles with the same dipole moment $\textbf{m}$ as the spherical particles at the same distance. View on the longitudinal axis cross-section of the sphere through the center ($z=0$).

By rearrangement of (33), the ratio of the magnetic dipole moment *m* and the magnetic dipole moment of a single particle in absence of the other particle $m_0=|{\mathrm{\alpha _m}} \textbf{H}_{\text{inc}}|$ can be expressed as a function of the distance *d*. Figure 14a shows the ratio $m/m_0$ obtained analytically with (33) as a function of the distance *d*. Furthermore, the numerically obtained solution of the ratio is plotted for the three distances from Fig. 13. Figure 14b shows $H_{\text{norm}}$ at the origin of the coordinate system from Fig. 13 obtained analytically with Eq. (5) and with numerical simulations at the distances from Fig. 13. It can be seen that the magnitude of $H_{\text{norm}}$ correlates with the ratio $m/m_0$. Furthermore, there is good agreement between the numerical and analytical results.

34 $$\begin{aligned} \frac{m}{m_0} = 1 - \frac{{\mathrm{\alpha _m}}}{4\pi d^3 + {\mathrm{\alpha _m}}} . \end{aligned}$$

#### Demagnetization formulas for prolate elliptical particles

The demagnetization factors of a prolate elliptical particles in the three Cartesian directions can be calculated with^58^

35 $$\begin{aligned}{} & {} N_z = \frac{1}{p^2-1}\left( \frac{p}{2(p^2-1)^{0.5}}\ln {\left( \frac{p+(p^2-1)^{0.5}}{p-(p^2-1)^{0.5}}\right) }-1\right) , \end{aligned}$$

36 $$\begin{aligned}{} & {} {N_x = N_y = \frac{p}{2(p^2-1)}\left( p-\frac{1}{2(p^2-1)^{0.5}}\ln {\left( \frac{p+(p^2-1)^{0.5}}{p-(p^2-1)^{0.5}}\right) } \right) }. \end{aligned}$$

#### Demagnetization formulas for oblate elliptical particles

The demagnetization factors of the oblate elliptical particles can be calculated with^58^

37 $$\begin{aligned}{} & {} N_z = N_x = \frac{1}{2(p^2-1)}(p^2(p^2-1)^{-0.5}\arcsin {((p^2-1)^{0.5}/p)}-1), \end{aligned}$$

38 $$\begin{aligned}{} & {} N_y = \frac{p^2}{p^2-1}\left( 1-\frac{1}{(p^2-1)^{0.5}}\arcsin {((p^2-1)^{0.5}/p)}\right) . \end{aligned}$$

## Data Availability

The datasets used and/or analysed during the current study is available from the corresponding author on reasonable request.

## References

[1] Patra JK (2018). Nano based drug delivery systems: Recent developments and future prospects. J. Nanobiotechnol..

[2] Mitchell MJ (2020). Engineering precision nanoparticles for drug delivery. Nat. Rev. Drug Discov..

[3] Cormode DP, Naha PC, Fayad ZA (2014). Nanoparticle contrast agents for computed tomography: A focus on micelles. Contrast Media Mol. Imaging.

[4] Alric C (2008). Gadolinium chelate coated gold nanoparticles as contrast agents for both X-ray computed tomography and magnetic resonance imaging. J. Am. Chem. Soc..

[5] Busquets MA, Estelrich J, Sánchez-Martín MJ (2015). Nanoparticles in magnetic resonance imaging: From simple to dual contrast agents. Int. J. Nanomed..

[6] Kaur P, Aliru ML, Chadha AS, Asea A, Krishnan S (2016). Hyperthermia using nanoparticles: Promises and pitfalls. Int. J. Hypertherm..

[7] Giustini AJ (2010). Magnetic nanoparticle hyperthermia in cancer treatment. Nano LIFE.

[8] Beik J (2016). Nanotechnology in hyperthermia cancer therapy: From fundamental principles to advanced applications. J. Controlled Release.

[9] Gavilán H (2021). Magnetic nanoparticles and clusters for magnetic hyperthermia: Optimizing their heat performance and developing combinatorial therapies to tackle cancer. Chem. Soc. Rev..

[10] Bisht G, Rayamajhi S (2016). ZnO nanoparticles: A promising anticancer agent. Nanobiomedicine.

[11] Huang X, El-Sayed MA (2010). Gold nanoparticles: Optical properties and implementations in cancer diagnosis and photothermal therapy. J. Adv. Res..

[12] Stetsenko MO (2017). Optical properties of gold nanoparticle assemblies on a glass surface. Nanoscale Res. Lett..

[13] Hammami I, Alabdallah NM, Jomaa AA, Kamoun M (2021). Gold nanoparticles: Synthesis properties and applications. J. King Saud Univ. Sci..

[14] Daniel M-C, Astruc D (2003). Gold nanoparticles: Assembly, supramolecular chemistry, quantum-size-related properties, and applications toward biology, catalysis, and nanotechnology. Chem. Rev..

[15] Kadhim RJ, Karsh EH, Taqi ZJ, Jabir MS (2021). Biocompatibility of gold nanoparticles: In-vitro and in-vivo study. Mater. Today Proc..

[16] Gu X (2020). Preparation and antibacterial properties of gold nanoparticles: A review. Environ. Chem. Lett..

[17] Shcherbakov V, Denisov SA, Mostafavi M (2023). A mechanistic study of gold nanoparticles catalysis of o2 reduction by ascorbate and hydroethidine, investigating reactive oxygen species reactivity. RSC Adv..

[18] Milan J, Niemczyk K, Kus-Liśkiewicz M (2022). Treasure on the earth: Gold nanoparticles and their biomedical applications. Materials.

[19] Yasin D (2022). Prospects in the use of gold nanoparticles as cancer theranostics and targeted drug delivery agents. Appl. Nanosci..

[20] Dreaden EC, Alkilany AM, Huang X, Murphy CJ, El-Sayed MA (2012). The golden age: Gold nanoparticles for biomedicine. Chem. Soc. Rev..

[21] Guglielmelli A (2021). Thermoplasmonics with gold nanoparticles: A new weapon in modern optics and biomedicine. Adv. Photon. Res..

[22] Terrés-Haro JM (2023). Finite element models of gold nanoparticles and their suspensions for photothermal effect calculation. Bioengineering.

[23] Chen J, Yuan M, Madison CA, Eitan S, Wang Y (2022). Blood-brain barrier crossing using magnetic stimulated nanoparticles. J. Control. Release.

[24] Georgas E, Yuan M, Chen J, Wang Y, Qin Y-X (2023). Bioactive superparamagnetic iron oxide-gold nanoparticles regulated by a dynamic magnetic field induce neuronal Ca2+ influx and differentiation. Bioactive Mater..

[25] Yuan M, Bancroft EA, Chen J, Srinivasan R, Wang Y (2022). Magnetic fields and magnetically stimulated gold-coated superparamagnetic iron oxide nanoparticles differentially modulate l-type voltage-gated calcium channel activity in midbrain neurons. ACS Appl. Nano Mater..

[26] Félix LL (2019). Gold-decorated magnetic nanoparticles design for hyperthermia applications and as a potential platform for their surface-functionalization. Sci. Rep..

[27] Wang P (2016). Assembly-induced thermogenesis of gold nanoparticles in the presence of alternating magnetic field for controllable drug release of hydrogel. Adv. Mater..

[28] Yoo J (2017). Electromagnetized gold nanoparticles mediate direct lineage reprogramming into induced dopamine neurons in vivo for parkinson’s disease therapy. Nat. Nanotechnol..

[29] Chang Y (2021). Electromagnetized gold nanoparticles improve neurogenesis and cognition in the aged brain. Biomaterials.

[30] Wei M, Yang Z, Li S, Le W (2023). Nanotherapeutic and stem cell therapeutic strategies in neurodegenerative diseases: A promising therapeutic approach. Int. J. Nanomed..

[31] Grant DN, Cozad MJ, Grant DA, White RA, Grant SA (2014). In vitro electromagnetic stimulation to enhance cell proliferation in extracellular matrix constructs with and without metallic nanoparticles. J. Biomed. Mater. Res. B.

[32] Mathematica’s Element Data function, I., Wolfram Research. Technical data for gold. https://periodictable.com/elements/079/data.html (2023).

[33] Amendola, V. *et al.* Physico-chemical characteristics of gold nanoparticles. In *Gold Nanoparticles in Analytical Chemistry*, 81–152. (Elsevier, 2014). 10.1016/b978-0-444-63285-2.00003-1.

[34] Li C-Y, Karna S, Wang C-W, Li W-H (2013). Spin polarization and quantum spins in Au nanoparticles. Int. J. Mol. Sci..

[35] Ayuela A, Crespo P, García MA, Hernando A, Echenique PM (2012). SP magnetism in clusters of gold thiolates. N. J. Phys..

[36] Ulloa JA (2021). Magnetism of dendrimer-coated gold nanoparticles: A size and functionalization study. J. Phys. Chem. C.

[37] van Rhee PG (2013). Giant magnetic susceptibility of gold nanorods detected by magnetic alignment. Phys. Rev. Lett..

[38] Trudel S (2011). Unexpected magnetism in gold nanostructures: Making gold even more attractive. Gold Bull..

[39] Nealon GL (2012). Magnetism in gold nanoparticles. Nanoscale.

[40] Gréget R (2012). Magnetic properties of gold nanoparticles: A room-temperature quantum effect. ChemPhysChem.

[41] Dong P, Fisher EA, Meli M-V, Trudel S (2020). Tuning the magnetism of gold nanoparticles by changing the thiol coating. Nanoscale.

[42] Guerrero E (2008). Surface plasmon resonance and magnetism of thiol-capped gold nanoparticles. Nanotechnology.

[43] Hurd C (1966). The magnetic susceptibility of silver and gold in the range 6–300 k. J. Phys. Chem. Solids.

[44] Evlyukhin AB, Tuz VR (2023). Electromagnetic scattering by arbitrary-shaped magnetic particles and multipole decomposition: Analytical and numerical approaches. Phys. Rev. B.

[45] Rochester university lecture notes chapter 6. http://teacher.pas.rochester.edu/phy217/lecturenotes/chapter6/lecturenoteschapter6.html.

[46] Landau, L. D. & Lifshitz, E. *Electrodynamics of Continuous Media (Course of Theoretical Physics)* (1984).

[47] Zywietz U, Evlyukhin AB, Reinhardt C, Chichkov BN (2014). Laser printing of silicon nanoparticles with resonant optical electric and magnetic responses. Nat. Commun..

[48] Tiwari P, Vig K, Dennis V, Singh S (2011). Functionalized gold nanoparticles and their biomedical applications. Nanomaterials.

[49] Sharma, R. G. The phenomenon of superconductivity. in *Superconductivity*, 13–48. (Springer, 2015). 10.1007/978-3-319-13713-1_2.

[50] Coey JMD (2010). Magnetism and Magnetic Materials.

[51] Erhardt JB (2018). Should patients with brain implants undergo MRI?. J. Neural Eng..

[52] Halchenko VY, Ostapushchenko DL, Vorobyov MA (2008). Mathematical simulation of magnetization processes of arbitrarily shaped ferromagnetic test objects in fields of given spatial configurations. Russ. J. Nondestruct. Testing.

[53] Beleggia M, Graef MD, Millev Y (2006). Demagnetization factors of the general ellipsoid: An alternative to the Maxwell approach. Philos. Mag..

[54] Rikken RSM (2014). Manipulation of micro- and nanostructure motion with magnetic fields. Soft Matter.

[55] Zablotskii V, Polyakova T, Lunov O, Dejneka A (2016). How a high-gradient magnetic field could affect cell life. Sci. Rep..

[56] Zablotskii V (2013). Life on magnets: Stem cell networking on micro-magnet arrays. PLoS ONE.

[57] Du D, Biswal SL (2014). Micro-mutual-dipolar model for rapid calculation of forces between paramagnetic colloids. Phys. Rev. E.

[58] Osborn JA (1945). Demagnetizing factors of the general ellipsoid. Phys. Rev..

